# Metastatic outgrowth encompasses COL-I, FN1 and POSTN upregulation and assembly to fibrillar networks regulating cell adhesion, migration and growth

**DOI:** 10.1186/1753-6561-6-S6-P39

**Published:** 2012-10-01

**Authors:** J Soikkeli, M Yin, E Hölttä

**Affiliations:** 1 Department of Pathology, Haartman Institute, University of Helsinki, Helsinki, Finland

## Background

Metastasis is a complex event in tumor development and accounts for most mortality in cancer patients. During this multistep process, cancer cells leave the primary site, intravasate into the blood or lymphatic vessels to disseminate, extravasate and finally outgrow at a distant site. Unfortunately, the outgrowth of metastatic cancer cells still remains poorly understood. It would therefore be important to unveil the molecular mechanisms involved in the development of micrometastases to overt metastases.

## Materials and methods

In the present study, we performed comparative DNA microarray (Affymetrix) analysis of melanoma lymph node micrometastases and macrometastases. Microarray data were analyzed by different methods and tools, including Significance Analysis of Microarrays (SAM) 3.0, fold change-ranking combined with *t* statistics (Volcano plot), GeneSpring GX 7.3, Gene Set Enrichment Analysis (Molecular Signature Database, v2.5), GenMAPP 2.1 and Ingenuity Pathway Analysis (IPA), to identify the genes and signaling pathways related to the outgrowth of metastases. The identified signaling pathways and genes were further validated by immunohistochemical and functional analyses.

## Results

We found that the transforming growth factor-beta (TGF-β) signaling pathway and TGF-β-induced extracellular matrix (ECM) molecules, periostin (POSTN), fibronectin 1 (FN1), collagen type I (COL-I) and versican (VCAN), were associated with the outgrowth of melanoma lymph node micrometastases to macrometastases. Immunohistochemical analyses confirmed the activation of TGF-β signaling pathway, as assessed by phospho-SMAD2 staining. The ECM proteins POSTN, FN1, COL-I and VCAN, together, were found to form fibrillar networks that regulated the adhesion, migration and growth of melanoma cells, fibroblasts and endothelial cells.

## Conclusions

The results suggest an import role for the TGF-β signaling pathway, and POSTN, FN1, COL-I and VCAN, in the regulation of metastatic outgrowth. Thus, TGF-β receptors and the metastasis-related matrix proteins FN1 and POSTN, in particular, may offer good therapeutic targets for the prevention of melanoma metastasis.

